# U-shaped relationship between current and pitch in helicene molecules

**DOI:** 10.1038/srep16731

**Published:** 2015-11-19

**Authors:** Yan-Dong Guo, Xiao-Hong Yan, Yang Xiao, Chun-Sheng Liu

**Affiliations:** 1 College of Electronic Science and Engineering, Nanjing University of Posts and Telecommunications, Nanjing 210046, China; 2 Key Laboratory of Radio Frequency and Micro-Nano Electronics of Jiangsu Province, Nanjing 210023, China; 3 China College of Science, Nanjing University of Aeronautics and Astronautics, Nanjing 210016, China

## Abstract

The helicene is constructed by twisted benzene or other aromatic rings, exhibiting a helical structure. Using first-principles calculations, we investigate the electronic transport of helicenes under stretching or compressing. Interestingly, a U-shaped curve of the current against *d* (the pitch of a helicene) is observed. Further analysis shows that, it is the result of the nonmonotonic change of HOMO-LUMO gap with *d*. The change of overlap between orbitals induced by conformational deformation is found to be the underlying mechanism. Moreover, the U-curve phenomenon is an intrinsic feature of the helicene molecules, being robust to the electrode materials or doping. This U-curve behavior is expected to be extended to helical graphene or other related structures, showing great application potential.

Graphene, a two-dimensional atomic crystal, has attracted much attention in recent years due to its unique electronic properties^1^,^2^,^3^,^4^. However, the practical application of it suffers from some intrinsic drawbacks, e.g., pristine graphene is a zero bandgap semiconductor^2^. To tune the electronic structures, kinds of methods based on structural transformation have been proposed^5^,^6^,^7^,^8^,^9^,^10^. It has been found a bilayer structure could open up a non-zero band gap, where the gap width would vary with the inter-layer distance^10^. Thus, inter-layer coupling is crucial to such carbon-based systems.

We here introduce a kind of molecules, helicenes^11^. They are constructed by twisted benzene or aromatic rings, exhibiting a helical structure. To some extent, they possess the structural features of both single-layer and bilayer (or multi-layer) graphene in a single molecule. Unlike traditional bilayer (or multi-layer) graphene structures, all the atoms of a helicene molecule reside geometrically in a single layer, and they interact with each other through not only intra-layer but also inter-layer interactions. In other words, the single (helical) layer also interact with itself through inter-layer interactions. Although many researches have been conducted on bilayer (or multi-layer) graphene or related structures, a system consisting of such a “tangled” coupling between intra-layer and inter-layer interactions is still lack of study.

It has been reported that, due to the inherently helical geometry, the *π*-conjugated framework is flexible and helicenes are able to act as molecular springs^11^,^12^,^13^,^14^,^15^. This means the inter-layer distance could be changed reversibly. As mentioned above, this distance is a regulator of the electronic properties in such a system, thus this feature shows huge possibilities for nanoelectronic device applications.

In this paper, we investigate the electronic transport of helicenes contacted with carbon chain or Au electrodes under stretching or compressing. To explore these two-probe systems more accurately, density functional theory calculations combined with nonequilibrium Green’s function are implemented^16^. Interestingly, a U-shaped curve of the current against *d* (the pitch of a helicene) is observed. Further analysis shows that, it is the result of the nonmonotonic change of HOMO-LUMO gap with *d*. And the change of overlap between orbitals induced by conformational deformation plays an important role. When revising this paper, we are aware of a recent theoretical work done by Vacek *et al.*^17^, which studies the conductance and thermopower of helicene molecules. They picked up one branch of the U-curve, obtaining the same conclusion that it is the orbitals’ overlap that is responsible to the variation of the current (or conductance)^17^. As their stretching is much smaller^17^, they do not see the increase in current for the stretched molecule, i.e., the right branch in our U curve. Moreover, we found the U-curve phenomenon is an intrinsic feature of the helicene molecules, being robust to the electrode materials or doping of heteroatoms. Due to the similarity in structure between helicene and graphene nanoribbon, this U-curve phenomenon is expected to be extended to helical graphene or other related structures, showing great application potential.

## Results

**Figure 1:**
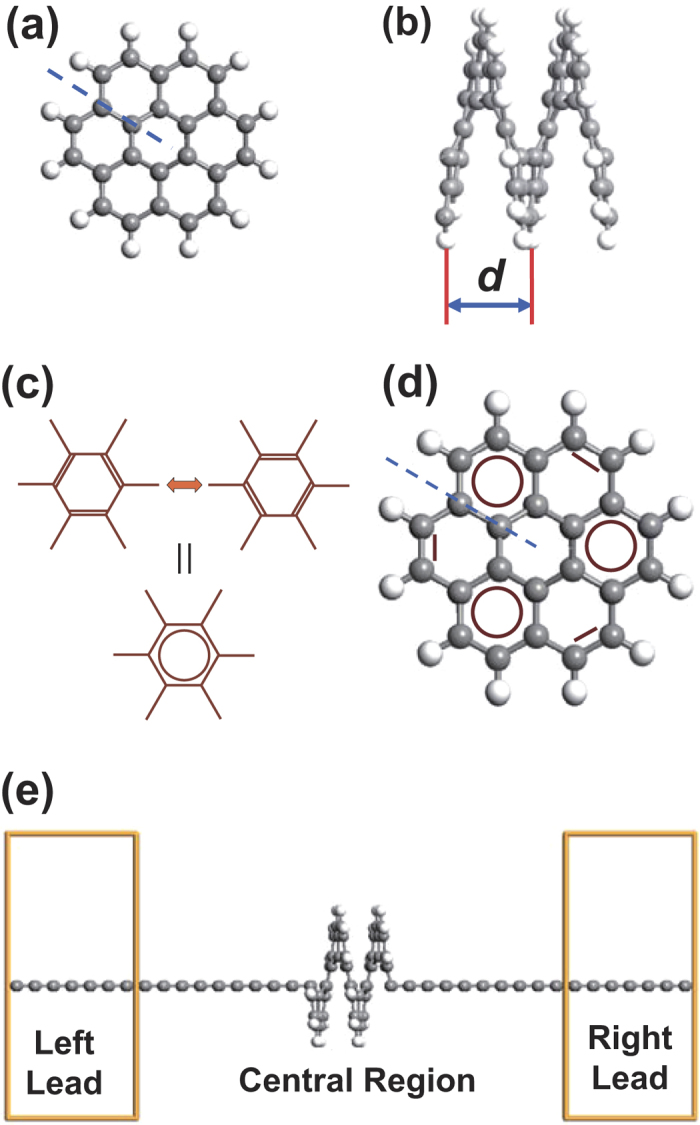
Structural properties of helicenes and related two-probe setup. (**a**) Axial and (**b**) side views of the [*n*]helicene (here *n* = 12) structure, where *n* is the number of aromatic rings in the helical molecule. The pitch between adjacent rings is denoted by *d*, as shown in (**b**). (**c**) Representation of the benzenoid carbon ring. (**d**) Schematic view of the bonds in [*n*]helicene molecules. (**e**) The setup of the two-probe system of carbon chain-helicene-carbon chain, where the semi-infinite carbon chain plays the role of electrodes (see more details in the Method section).

Helicene molecules are polycyclic aromatic compounds, which are constructed by ortho-fused benzene (or other aromatic) rings^11^,^18^,^19^. The arising of nonplanar structure is due to the twisted ring-ring connections. As the number of aromatic rings increases, the molecule spirals up along the spiral axis, forming a nonplanar screw-shaped structure. We could name a helicene molecule by adding a bracket [*n*] with a number *n* before the helicene, e.g., [5]helicene^19^,^20^. And *n* represents the number of aromatic rings in the helical molecule. The helicene has a constant pitch for both inner and outer helixes, denoted as *d* in this work, see Fig. 1(b). If a helicene molecule is constructed by sixmembered aromatic rings (like benzene), nearly six rings will cover a rotation of 360°^ ^21, as shown in Fig. 1(a,b). According to the helicity rule proposed by Cahn *et al.*^22^, the helicene we studied here, shown in Fig. 1(a,b), is a (*P*)-helicene, which means it is a right-handed helix and denoted by 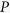 (stands for plus).

We now pay attention to the stability of such a helical structure, which is a crucial issue to the practical application. In hydrocarbon structures, every carbon atom should possess 4 saturated bonds, which is the criterion of structural stability^23^. As *π* electrons are delocalized over the ring^24^, a benzenoid aromatic ring could be defined as a resonance between two sixmembered hexagonal rings which have alternating single and double C-C bonds^23^, as shown in Fig. 1(c). According to the well-known Clar’s rule^23^,^25^,^26^, for aromatic hydrocarbons, the stability of the isomer increases with the number of possible resonances. In other words, the stability increases with the number of possible resonances. Graphene shows good stability, as 1/3 rings in it could be benzenoid ones^23^. For our helicenes, this ratio becomes 1/2, see Fig. 1(d), so helicene molecules have high stability.

In this work, in order to explore the intrinsic feature of the helicene, we choose carbon linear atomic chains to be the electrodes, as they can strip off the complications which may arise from electrode-molecule interactions (in the following, Au electrodes are also used for some cases for comparison purposes). Carbon chain electrodes have been adopted in many previous studies, because they are metallic and could make good contact with organic molecules^27^,^28^. The whole setup of the two-probe system is shown in Fig. 1(e). The left/right electrode is semi-infinite, which is represented by the left/right electrode cell. The central region consists of the helicene molecule and the left/right electrodes extension, which are also called screening regions. The left and right electrodes are kept at fixed potentials, and the potential drop occurs in the central region. The Hamiltonian of the central region depends on the nonequilibrium electron density and is calculated through a self-consistent procedure, where the influence of electrodes is taken into account by the self-energies^16^.

As far as we know, the longest helicene that has been discovered is [25]helicene^11^. The convergence of the physicochemical properties, including stability and relative changes in energy of the structures as well as molecular orbital localizations, with the number of aromatic rings (*n*) has been systematically investigated by Rulíšek *et al.*^29^. Balancing the computation cost and molecule length, [12]helicene consisting of 12 benzene rings is adopted in this work. It covers a rotation of 720°, ensuring the existence of an inter-layer interaction for each part of the molecule.

**Figure 2:**
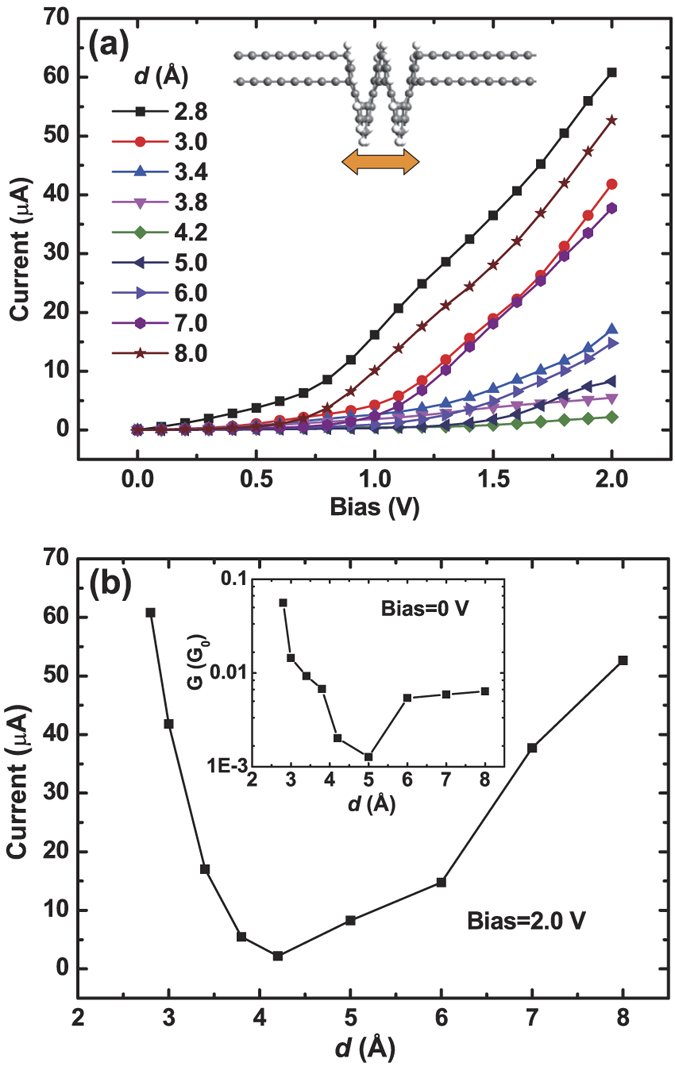
*I* − *V* behaviors of [12]helicene contacted with carbon chain electrodes. The pitch of the relaxed (gas-phase) [12]helicene molecule is *d* = 3.4 Å. (**a**) *I* − *V* curve under different pitch *d*. (**b**) Current varies with *d* under the bias of 2.0 V. Inset: Conductance under zero bias on a logarithmic scale. *G*_0_ is the conductance quantum (*G*_0_ = 2*e*^2^/*h*).

**Figure 3:**
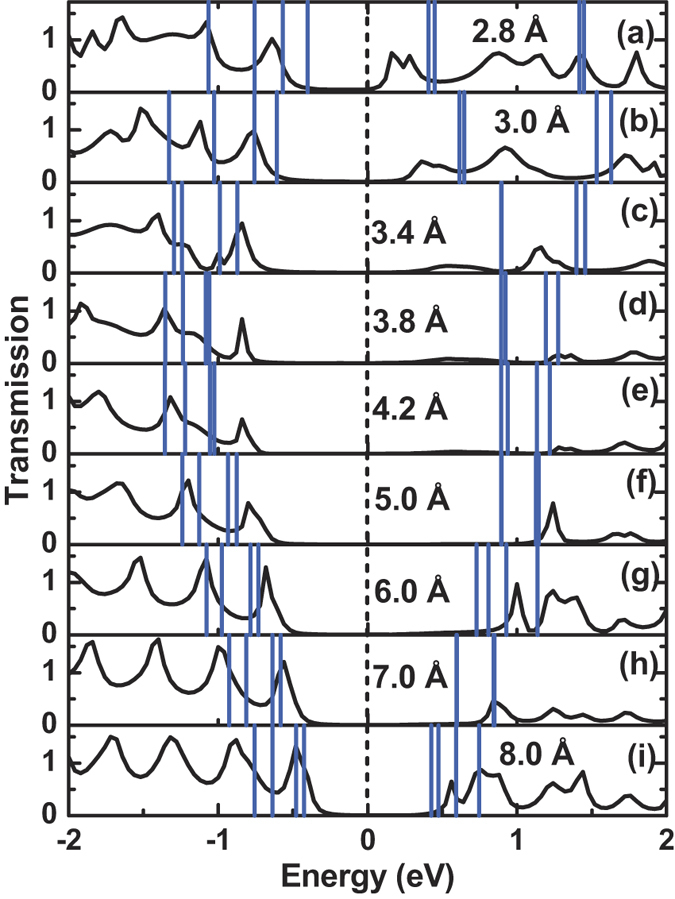
(**a**–**i**) Transmission spectra of [12]helicene contacted with carbon chain electrodes under zero bias for different pitch *d*, which is denoted in the figure. For each case, the molecular orbital levels around the Fermi energy in the isolated molecule of [12]helicene are presented, i.e., HOMO, LUMO, HOMO-N, and LUMO + N (N = 1, 2 and 3). The Fermi energy is set to be zero. Note that the transmission spectra are obtained under zero bias. We also calculate the spectra under other non-zero biases, and only negligible changes are observed.

The molecule has been optimized before transport calculations. The pitch of the relaxed (gas-phase) [12]helicene molecule is *d* = 3.4 Å, which is close to the inter-layer distance in bilayer graphene nanoribbons (3.2 Å)^10^. When contacted with carbon chain electrodes, the *I* − *V* (current-bias) curve of it is calculated and shown in Fig. 2(a). One finds in this curve (*d* = 3.4 Å) there is a threshold bias voltage of 0.5 V. When the bias is less than 0.5 V, the current is almost zero. Once the bias is larger than 0.5 V, the current will increase as the bias goes up. This *I* − *V* behavior could be interpreted through the electron transport spectrum shown in Fig. 3(c). Apparently, around the Fermi energy (the Fermi level is set to be zero in this work), the transmission is nearly zero. Around 0.5 eV, there is a small transmission peak. According to Eq. (1), the current is determined by the integral area in the bias window. So, once this transmission peak enters into the integral window, there will be a non-zero current. To be specific, the left edge of the peak is at about 0.25 eV, thus it will enter into the bias window ([−0.25, 0.25] V) and contributes to the current when bias ≥0.5 V. If the bias increases further, more transmission peaks will take part in the integration and the current goes up continuously, just like the bias ≥0.5 V cases in Fig. 2(a) (*d* = 3.4 Å).

Previous studies show that helicene framework is flexible and can be used as springs^11^,^12^,^13^,^14^,^15^. We next compress or stretch the molecule (see the Method section for optimization details), and calculate the corresponding *I* − *V* curves. Compressing or stretching will change the pitch *d* (inter-layer distance) in the molecule, which is crucial to such a system. As both intra- and inter-layer interactions exist in a single helical layer, an unusual transport behavior is expected. Figure 2(a) shows the *I* − *V* curves for a series of *d*, i.e., 2.8, 3.0, 3.4, 3.8, 4.2, 5.0, 6.0, 7.0 and 8.0 Å. One finds the transport of the molecule undergoes a dramatic change as *d* varies. For instance, the threshold bias voltage changes quite a lot. From *d* = 2.8 to 4.2 Å, this voltage increases, e.g., zero for *d* = 2.8 Å and 0.5 V for *d* = 3.4 Å. Interestingly, from *d* = 4.2 to 8.0 Å, this voltage decreases, e.g., 1.3 V for *d* = 5.0 Å and 0.5 V for *d* = 8.0 Å. Apparently, *d* = 4.2 is a turning point. The transport behaviors are quite different between *d* < 4.2 and *d* > 4.2 Å cases. To see this behavior more clearly, we choose the condition under bias = 2.0 V for further studies. The current varies with *d* under bias = 2.0 V is plotted in Fig. 2(b). From this figure, one finds as *d* increases from 2.8 to 8.0 Å, the current decreases first and then increases, exhibiting a distorted “U” shape. The bottom of the U-shaped curve corresponds to the *d* = 4.2 Å case, which has the smallest current. To compare with the studies of Vacek *et al.*^17^, we also show the variation of the zero-bias conductance in the inset of Fig. 2(b). The left branch of it (*d* ≤ 5.0 Å) agrees well with the result obtained by Vacek *et al.*^17^ (they only picked up this branch). Note that bulk electrode in their studies would provide more transmission channels than nano-electrode and result in a larger conductance^17^. As the main transmission peaks are not within the bias window at a lower bias, the shape of the zero-bias U curve is not exactly the same as that of bias = 2.0 V case.

This U-curve behavior is different from that of other carbon-based systems. Farajian *et al.*^30^ found, at a finite bias, the current of semiconducting tube increases with increased bending, while that of metallic tube decreases. Although they exhibit inverse variations of currents, it is not realized in a single structure like helicene. In parallel nanotube junction, Xu *et al.*^31^ found the conductance exhibit various behaviors as a function of the contact length, which is the result of quantum interference of Bloch waves. However, different from that structure, our system lacks periodicity. In bilayer graphene nanoribbons, Paulla *et al.*^32^ reported that, the bandgap is shown to depend on relative orientation of the two layers. For our systems, we checked the stacking orientation and found almost no change under compressing and stretching. This might be due to the “end-fixed” optimization method, which actually satisfies the requirement of practical applications. It is also found that, in bilayer graphene devices, as the inter-layer distance increases, the bandgap increases monotonously and the current decreases^10^. The helicene molecule is also a carbon-based structure constructed by aromatic rings, so it is interesting to investigate the physical mechanisms in the U-shaped transport behavior.

Figure 3(a–i) show the transmission spectra for different *d* (denoted in each figure), which correspond to the points in Fig. 2(b) respectively. Around the Fermi energy, there is a transmission gap. In the *d* = 4.2 Å case, the gap is the largest. From *d* = 4.2 to 2.8 Å, i.e., from Fig. 3(a–e), the gap decreases, and a transmission peak above zero energy arises and moves to the Fermi level. From *d* = 4.2 to 8.0 Å, i.e., from Fig. 3(e–i), the transmission gap also decreases monotonously. Obviously, it is these changes of transmission gap with the variation of *d* that result in the U-curve behavior.

To address the underlying origins for the transmission gap, especially for the U-curve behavior, we calculate the molecular orbital (MO) levels of the helicene. By contacting with electrodes, these MO levels will be broadened and facilitate the transmitting of electrons. For each case in Fig. 3, eight MO levels around the Fermi energy in the isolated molecule of [12]helicene are plotted (vertical and blue lines), i.e., highest occupied molecular orbital (HOMO), lowest unoccupied molecular orbital (LUMO), HOMO-N, and LUMO + N (N = 1, 2 and 3). It is these orbitals that contribute to the transmissions around the Fermi level. From the figures, one finds as *d* increases from 2.8 to 8.0 Å, the HOMO-LUMO gap also increases first and then decreases. It is in line with the variation of transmission gap. Therefore, it is the nonmonotonic change of HOMO-LUMO gap with *d* that results in the U-curve behavior of the *I* − *d* relation.

Note that the HOMO-LUMO gap of the helicene is obtained without connection to electrodes. The procedure is as follows. We first optimize the constrained isolated molecules. Then, we calculate the electronic structure of these isolated molecules and obtain the HOMO-LUMO gap. After that, the molecules are connected to the electrodes to calculate the transport properties.

Vukmirovic *et al.*^33^ reported the energy gap of bent thiophene chains changes depending on the bending angle. The change of overlap between orbitals induced by conformational deformation is the underlying mechanism, which we will show in the following is in consonance with that of our systems.

For *π*-conjugated molecule, the effect of torsion has been widely studied^34^,^35^. It will cause the decrease of overlap of *π* orbitals between neighboring rings. When the torsion is large enough, the extent of the wave function will be broken. As a result of quantum confinement effect, the torsion increases the energy gap^33^. Based on this feature, kinds of nanoelectronic devices have been proposed, e.g., switch and logic operators^36^,^37^.

On the contrary, bending would decrease the energy gap, which has been demonstrated in thiophene chains^33^. This is because bending will increase the overlap of *π* orbitals between neighboring rings, and tend to localize the wave function around the bending region. As proposed by Vukmirovic *et al.*^33^, the effect of bending can be seen as a defect which creates a localized state in the energy gap.

**Figure 4:**
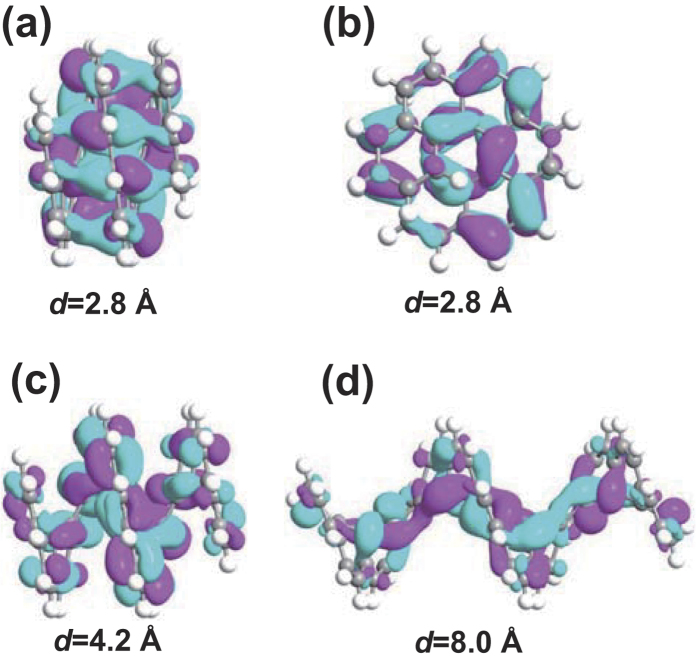
Geometries and spatial distributions of LUMO states for isolated [12]helicene. The isovalue is 0.02 Å^−3/2^. (**a**) Side and (**b**) axial views of *d* = 2.8 Å case. (**b**) *d* = 4.2 Å case. (**c**) *d* = 8.0 Å case.

In both torsion and bending effects, one finds the strength of orbitals’ overlap is the key mechanism. From Fig. 3, we can see for our system LUMO is the nearest orbital to the Fermi level. In other words, LUMO has a large influence on the electronic transport. So we take LUMO as an example to see the effect of the change of orbital’s overlap on the transport. Figure 4 shows the geometries and spatial distributions of LUMO states of isolated helicenes for three special cases, i.e., *d* = 2.8, 4.2 and 8.0 Å.

For the left branch of our U curve, i.e., *d* = 2.8 ~ 4.2 Å, the current increases with the decrease of *d*. According to the mechanism of orbitals’ overlap, there should be an increase of overlap. In Fig. 4(a), one finds there are large overlap of orbitals between adjacent layers. Easy to know, the smaller the *d* is, the larger the overlap becomes. As a consequence, the current increases when *d* becomes smaller. Comparing Fig. 4(a,b), we can see the inter-layer overlap is even larger than that of intra-layer ones. Note that these two figures, Fig. 4(a,b), are plotted with the same isovalue. As *d* decreases, the wave function tends to localize in the inter-layer regions, which contributes the transmission channels in the two-probe systems. Like the thiophene chain cases, to some extent, the effect of compressing could be understood as the defect which creates an inter-layer localized state in the energy gap, resulting in the decrease of it. Therefore, we can conclude inter-layer transmission is dominant in the left branch of the U curve.

For the right branch of the U curve, i.e., *d* = 2.8 ~ 4.2 Å, the current increases with the increase of *d*. As a representative of this branch, Fig. 4(d) shows the spatial distribution of LUMO state for *d* = 8.0 Å case. From the figure, one finds stretching tends to localize the wave function toward the inner edge region. Similarly, the stretching can be seen as the defect that creates an intra-layer localized state in the energy gap, also resulting in the decrease of it. Thus, in the right branch of the U curve the intra-layer transmission is dominant.

When the molecule is stretched, the bonding between neighboring atoms should be weaker and weaker. It is interesting to figure out why the total current increases under this circumstance. Actually, this is reasonable. When we stretch the helicene, the torsion of the inner part, especially the inner edge, becomes smaller. One can image the limit of stretching, where the inner edge will become a linear chain (the molecule might be totally destroyed before reaching it). Smaller torsion indicates larger overlap of orbitals. This can be seen in Fig. 4(d). To some extent, a graphene nanoribbon-like “inner edge state” is created. It contributes to the transmission channels for the intra-layer transport.

**Figure 5:**
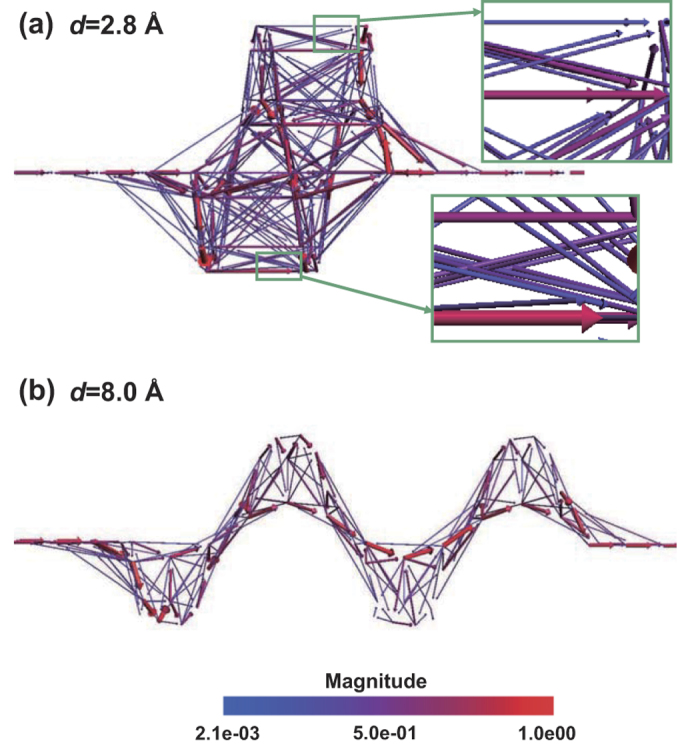
Transmission pathways of [12]helicene contacted with carbon chain electrodes. The volume and color of the arrow illustrate magnitude of the pathway. (**a**) for *d* = 2.8 Å case and (**b**) for *d* = 8.0 Å case. (**c**) Take the *d* = 2.8 Å case as an example to show the two-probe structure.

Moreover, we calculate the transmission pathways, through which we could observe the transmission in three-dimensional real space. In transmission pathway analysis, the transmission coefficient is split into local bond contributions^38^. The *d* = 2.8 and 8.0 Å cases are the tails of the two branches of the U curve. We here take them as the representatives of two regimes to show the pathways of the transmission, shown in Fig. 5(a,b), respectively. In order to see the pathways more clearly, the balls representing the atoms and the sticks representing the bonds are not plotted. In the *d* = 2.8 Å case, see Fig. 5(a), one finds there are both intra- and inter-layer transmissions. But in the *d* = 8.0 Å case, shown in Fig. 5(b), only intra-layer transmission exists. Figure 5(a,b) clearly show the transition between the two transport regimes.

In short, the two branches belong to two transport regimes respectively, i.e., inter-layer transmission and intra-layer transmission. It should be noted that, *d* = 4.2 Å is a “critical point”, where neither of them is dominant. This U-curve behavior is expected to be extended to helical graphene or other related structures, showing great application potential. However, the edges and width are crucial to the gap of a graphene nanoribbon. And it will be more complicated for a twisted one. If we extend our system to graphene-based structures, these parameters have to be considered. In this work, we confine our research to the simplest structure, i.e., helicene, which has been synthesized experimentally. We hope to do a systematic study on it in the future.

## Discussion

**Figure 6:**
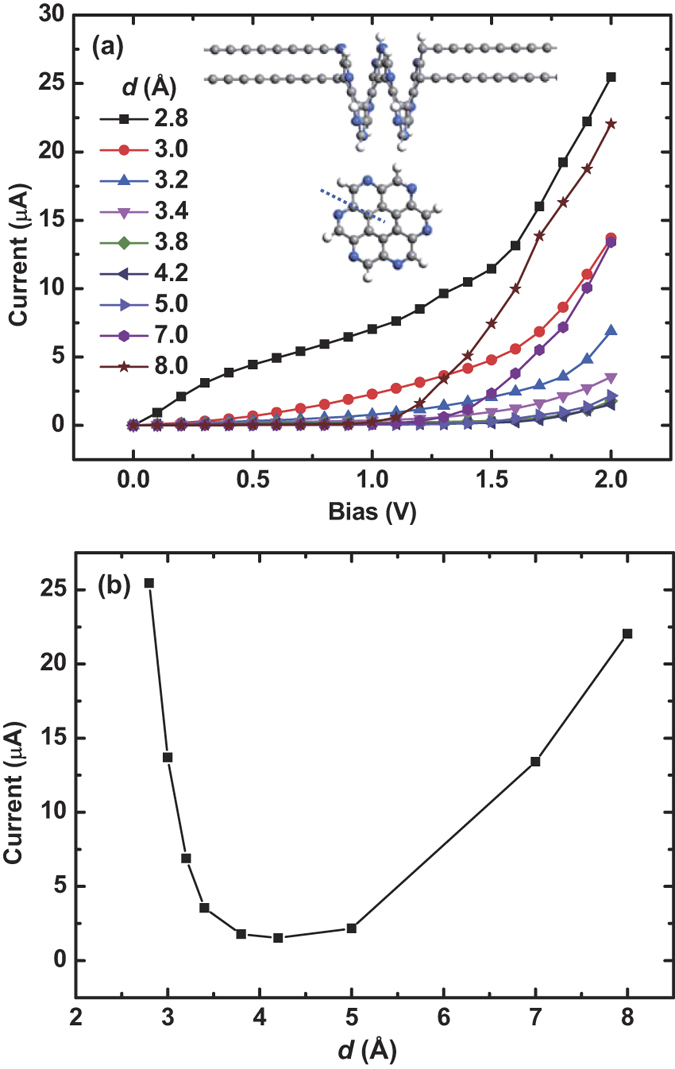
*I* − *V* behaviors of aza[12]helicene contacted with carbon chain electrodes. (**a**) *I* − *V* curve under different pitch *d*. As shown in the inset, aza[12]helicene is composed of pyridines. (**b**) Current varies with *d* under the bias of 2.0 V.

The [12]helicene molecule we studied above is a carbohelicene, which is composed solely of benzene rings. There are also heterohelicenes, which contain at least one heteroatom in the framework. In order to investigate the doping effect on the U-curve phenomenon, we choose a heterohelicene molecule, aza[12]helicene, to study its *I* − *V* behavior. Aza[12]helicene is composed of pyridines, shown in the inset of Fig. 6(a). Figure 6(a) shows the *I* − *V* curves for different *d*, and Fig. 6(b) shows the *I* − *d* relation under the bias of 2.0 V. Compared with the [12]helicene cases, the currents of aza[12]helicene systems are suppressed due to the doping of N atoms. However, the U-curve behavior still exists, implying it is an intrinsic feature of such helical molecules and robust to the doping of heteroatoms.

**Figure 7:**
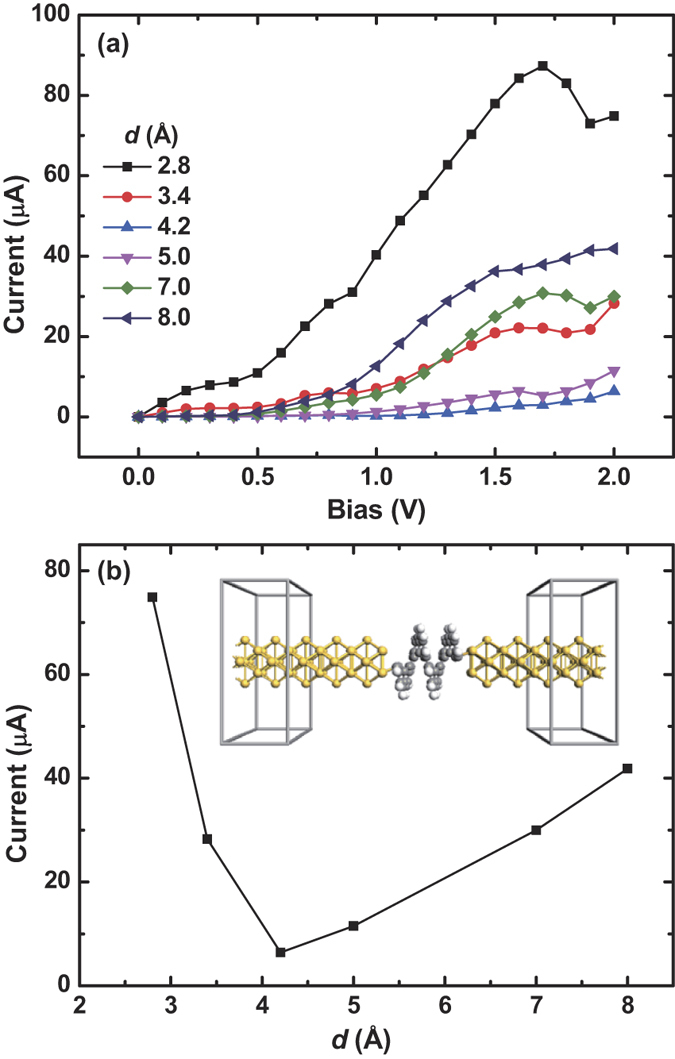
*I* − *V* behaviors of [12]helicene contacted with Au electrodes. (**a**) *I* − *V* curve under different pitch *d*. (**b**) Current varies with *d* under the bias of 2.0 V.

In order to investigate the effect of electrode materials on transport behaviors, we also calculate the *I* − *V* curves for the [12]helicene contacted with Au electrodes for different *d*, shown in Fig. 7(a). Au electrode is one of the most commonly used electrode structures in nano-electronics. Due to the large computation cost induced by the increase of atom number, fewer pitches are chosen to calculate the *I* − *V* curves. Figure 7(b) shows the *I* − *d* relation under the bias of 2.0 V. From the results, one finds the U-curve feature still exists. It is indeed an intrinsic feature of such a helical structure, being robust to doping or electrode materials. Surprisingly, negative differential resistance (NDR) behavior also emerges, see *d* = 2.8 and 7.0 Å cases, which is very useful in nano-electronic devices. This phenomenon may be caused by kinds of mechanisms, e.g., molecule-electrode coupling, charge transfer, bias induced changes in molecular orbitals, and molecular orbitals alignment^39^,^40^,^41^,^42^. In our calculations, the potential drop is calculated self-consistently (using the Possion equation), avoiding the drawback in phenomenological models for NDR^39^. However, this is beyond the scope of this paper and we wish to investigate in future studies.

It should be noted that we do not optimize the contact geometry. When the stretched (or compressed) helicene molecules have been fully optimized, we contact them to the electrodes by choosing a reasonable electrode-molecule distance, which is taken from literature^43^. Such a contact method is commonly used in “Au-organic molecule-Au” systems for transport studies^44^,^45^. In practical applications, the geometry of contact is crucial, which needs a systematic investigation. In this work, what we try to demonstrate is that, the alignment of energy levels in helicene and Au electrodes does not destroy the U-shaped feature. For a system like ours, this is also essential to the practical applications.

Another important issue is at what length the molecule will detach from the electrodes when stretching it. The corresponding detaching pitch should be larger than 4.2 Å in order to observe the U curve in experiment. It has been reported that a high energy of 3.0 eV for rupturing Au-C bond is observed in “Au-organic molecule-Au” single-molecule junctions^46^. According to this energy and the structural parameters (not shown) of the stretched molecule, a rough estimate of the detaching pitch would be 7.0 ~ 8.0 Å (9.0 Å is found to be the stretching limit of our helicene molecule), which is larger than 4.2 Å. Actually, the observation of the U curve could also be ensured by connecting the molecule to nanotube or graphene through covalent C-C bond. These structures (nanotube and graphene) can form strong contacts with metal electrodes, or they can be used as electrodes themselves.

In summary, based on the density functional theory combined with nonequilibrium Green’s function, we preformed a first-principles calculation on the electronic transport of helicenes under stretching and compressing. A U-shaped curve of *I* − *d* (the current against the pitch) is observed. Further analysis shows that, it is caused by the nonmonotonic change of HOMO-LUMO gap, as well as the transmission gap, with *d*. The change of overlap between orbitals induced by conformational deformation is found to be the underlying mechanism. And a transition of electronic transport between inter- and intra-layer transmission-dominated regimes is observed. Moreover, the systems of C-aza[12]helicene-C and Au-[12]helicene-Au are also investigated, and the U-curve behavior remains, being robust to doping of heteroatoms or the electrode materials. This implies the U-curve phenomenon is an intrinsic feature of such helical molecules, and it has a wide range of applications in nano-electronic devices.

## Methods

Based on the combination of density functional theory (DFT) and nonequilibrium Green’s function (NEGF), the transport properties are investigated by the first-principles calculations, which are carried out through Atomistix Toolkit package^16^,^42^. The DFT + NEGF method has been widely used in electronic transport calculations for nanostructures^47^,^48^. The results of it are in good agreement with the experiments and the calculations using other methods for a wide range of systems^16^. We use the mesh cutoff energy of 150 Ry and 1 × 1 × 100 *k*-point mesh in the Monkhorst-Park scheme^49^. Double-zeta polarized basis set of the local numerical orbitals and Perdew-Burke-Eenzerhof (PBE) formulation to the generalized gradient approximation (GGA) are employed^50^. To eliminate the interactions with adjacent images, sufficient vacuum spaces (more than 10 Å) are included in the supercell. The above parameters we choose have been widely adopted in transport calculations in such kind of systems^44^,^51^. The reliability of them have been tested both in theory and experiment^16^,^48^,^52^.

Before transport calculations, the helical molecules are optimized as follows. We first change the coordinates of all the atoms in the molecule along the spiral axis proportionally, in order to achieve a specific length for the molecule. Then, we fix the positions of the end atoms of the molecule to mimic the compression or stretch, and let the rest of atoms be fully optimized until all the forces are less than 0.02 eV/Å. In other words, the constraints are applied by controlling the length of the molecule.

After optimizing the stretched (or compressed) helicene molecules, we contact them with the electrodes without geometric optimization for the contact region, where the electrode-molecule distance is taken from literature^27^,^28^,^53^. Such a contact method is often used in electronic transport studies^44^,^45^.

For practical applications, the geometry of contact is crucial, especially under constraints. This would need a systematic study. Here, we focus on the intrinsic transport feature of the helicene molecule. The carbon chain in our system is more like a model electrode, as it is metallic and could make good contact with organic molecules^27^. From the results, one finds the contact factor has less impact on the performance of the U-shaped feature.

In the NEGF + DFT scheme, a current is obtained through the Landauer-Büttiker formula


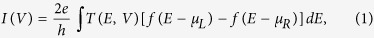


where *f*(*E* − *μ*_*L*/*R*_) and *μ*_*L*/*R*_ are the Fermi function and chemical potential in the left/Right electrode, respectively. The bias window between two electrodes is *V* = (*μ*_*L*_ − *μ*_*R*_)/*e*. Transmission probability of an electron *T*(*E*, *V*) is obtained according to


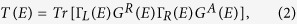


where Γ_*L*/*R*_ is the coupling matrix between the molecule and the electrode, and *G*^*R*/*A*^ is the retarded (or advanced) Green’s function.

For a two-probe system, the analysis of transmission pathway could help us explore the local current. The method we adopted is proposed by Solomon *et al.*^38^. The essential consideration is the conservation of charge, i.e., if the system is divided into two parts (*I*, *J*) by an arbitrary surface perpendicular to the transport direction, the sum of the local currents through the surface should equal the total current.


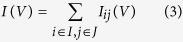


The sum runs over all pairs of atoms *i* and *j*, which belong to *I* and *J* respectively.

In the case of transmission, the relationship would be


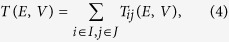


where the transmission coefficient is split into local bond contributions. In this paper, the magnitude of the pathway is illustrated by both the volume and color of the arrow.

## Additional Information

**How to cite this article**: Guo, Y.-D. *et al.* U-shaped relationship between current and pitch in helicene molecules. *Sci. Rep.* **5**, 16731; doi: 10.1038/srep16731 (2015).
